# Measured and genetically predicted protein levels and cardiovascular diseases in UK Biobank and China Kadoorie Biobank

**DOI:** 10.1038/s44161-024-00545-6

**Published:** 2024-09-25

**Authors:** Lars Lind, Mohsen Mazidi, Robert Clarke, Derrick A. Bennett, Rui Zheng

**Affiliations:** 1 Department of Medical Sciences, Uppsala University, Uppsala, Sweden; 2 Nuffield Department of Population Health, University of Oxford, Oxford, UK

**Keywords:** Translational research, Stroke, Proteomics, Myocardial infarction, Heart failure

## Abstract

Several large-scale studies have measured plasma levels of the proteome in individuals with cardiovascular diseases (CVDs)^1–7^. However, since the majority of such proteins are interrelated^2^, it is difficult for observational studies to distinguish which proteins are likely to be of etiological relevance. Here we evaluate whether plasma levels of 2,919 proteins measured in 52,164 UK Biobank participants are associated with incident myocardial infarction, ischemic stroke or heart failure. Of those proteins, 126 were associated with all three CVD outcomes and 118 were associated with at least one CVD in the China Kadoorie Biobank. Mendelian randomization and colocalization analyses indicated that genetically determined levels of 47 and 18 proteins, respectively, were associated with CVDs, including FGF5, PROCR and FURIN. While the majority of protein–CVD observational associations were noncausal, these three proteins showed evidence to support potential causality and are therefore promising targets for drug treatment for CVD outcomes.

## Abstract (web summary)

Lind et al. investigate the causal relationship between plasma proteins and cardiovascular disease outcomes in patients of European and Chinese descent, identifying FGF5, PROCR and FURIN as promising targets for the development of new drugs.

## Main

Several studies have measured protein plasma levels in individuals with cardiovascular disease (CVD) outcomes^1–7^. However, since the majority of proteins are interrelated^2^, it is difficult for observational studies to identify proteins of etiological relevance. One way to address this problem is to use instrumental variables to investigate whether genetic loci regulating plasma levels of individual proteins also are related to different CVD outcomes. Here, we used observational and genetic analyses to assess the associations of up to 2,919 proteins with risk of myocardial infarction (MI), ischemic stroke (IS) or heart failure (HF) or a combination of all such diseases in the UK Biobank (UKB) to discover novel targets for drug treatment. Overall, 636 of those proteins were related to any CVD outcome and 126 were associated with all three CVDs. Of those, 118 were replicated to be associated with at least one CVD outcome in the China Kadoorie Biobank (CKB). Mendelian randomization (MR) and colocalization analyses indicated that genetically determined levels of 47 and 18 proteins, respectively, were associated with CVDs, including fibroblast growth factor 5 (FGF5), protein C receptor (PROCR) and FURIN. While the majority of protein–CVD observational associations were noncausal, these three proteins showed evidence of potential causality and are therefore promising targets for drug treatment for CVD outcomes.

For the observational part, we used plasma levels of 2,919 proteins measured in 52,164 UKB participants. Selected baseline characteristics of this sample are provided in Extended Data Table 1. In this sample, 1,345 experienced a first MI, 934 a first IS and 1,971 received a diagnosis of HF for the first time during a median follow-up of 12.6 years. In the discovery subsample (a random two-thirds of the sample), 1,105 proteins were significantly associated with any CVD outcome at a false discovery rate (FDR) of <0.05. When these proteins were evaluated in the internal validation subsample (the remaining one-third) in a similar fashion, 636 proteins showed an FDR of <0.05. The top five most strongly associated proteins were ADM, PGF, SHISA5, WFDC2 and PLAUR. The hazard ratios (HRs) were in the 2.5–3.5 range for a 1 s.d. change in protein levels (Supplementary Table 1).

In a pathway enrichment analysis of the genes for those proteins, pathways related to inflammation and extracellular matrix dominated. In addition, regulation of insulin-like growth factor (IGF) transport, posttranslational protein phosphorylation, integrin surface interactions and growth factors were among the enriched pathways (Supplementary Table 2).

**Fig. 1:**
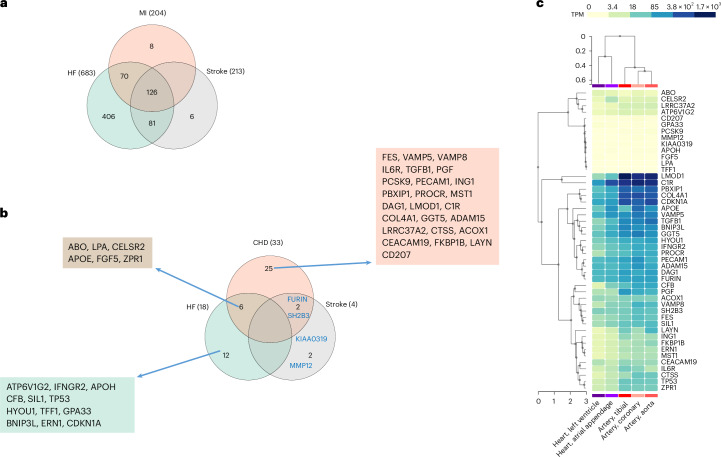
Numbers of proteins associated with the three CVD outcomes and gene expression level look-up. **a**, A Venn diagram visualizing the overlap of measured protein levels associated with incident CVDs by Cox proportional hazards regression after Bonferroni correction (*P* < 0.000017, two sided). Only the numbers of proteins are given. For details, see Supplementary Tables 6–8. **b**, A Venn diagram visualizing the overlap of genetically predicted levels of proteins associated with the three CVDs (CHD, IS and HF) on MR analysis (Wald ratio method). Only proteins with an FDR <0.05 (two sided) are shown. **c**, The expression levels of genes coding proteins found to be significant by MR analysis were looked up in human heart and artery tissues on the GTEx Portal. TPM, transcripts per million.

In the analysis of the complete sample, 204 proteins were related to MI (Fig. 1a) at *P* < 0.000017 (Bonferroni adjusted). The top five proteins associated with MI were PLAUR, EDA2R, WFDC2, IGFBP4 and GDF15 (Supplementary Table 3). Likewise, 213 proteins were related to IS (Supplementary Table 4) with the top five hits being CKAP4, WFDC2, NPC2, IGFBP4 and NEFL, while 683 were related to HF with the top five hits being ADM, COL18A1, ACVRL1, CRIM1 and EDN1 (Supplementary Table 5). An overview is provided in Supplementary Tables 6 and 7.

**Extended Data Fig. 1:**
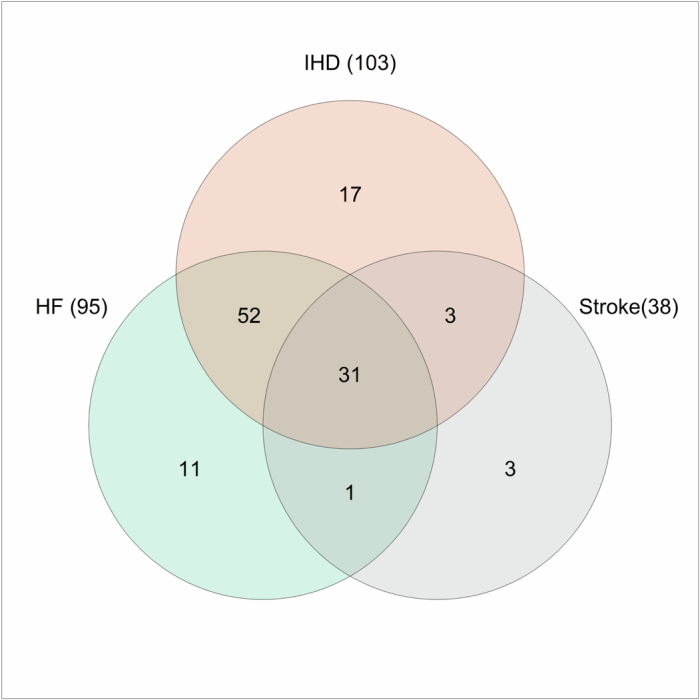
Numbers of proteins of interest associated with three CVD outcomes in CKB. Venn diagram visualizing the overlap of measured protein levels significantly (nominal *P* < 0.05) associated with incident cardiovascular diseases (ischemic heart disease (IHD), ischemic stroke; and heart failure, HF) in the replication phase in China Kadoorie Biobank (CKB) by Cox proportional hazards regression. Only the numbers of proteins are given. Source data can be found in Supplementary Table 8.

A total of 126 of the proteins were related to all three CVD outcomes in UKB. Of those, 118 were related to any of the CVDs in the replication phase in CKB, when the same proteins were related to incident cases of the three CVDs in 1,937 participants (Extended Data Fig. 1 and Supplementary Table 8). Overall, 87 of the proteins were related to more than one of the three CVD traits and 31 were related to all three traits at *P* < 0.05.

**Fig. 2:**
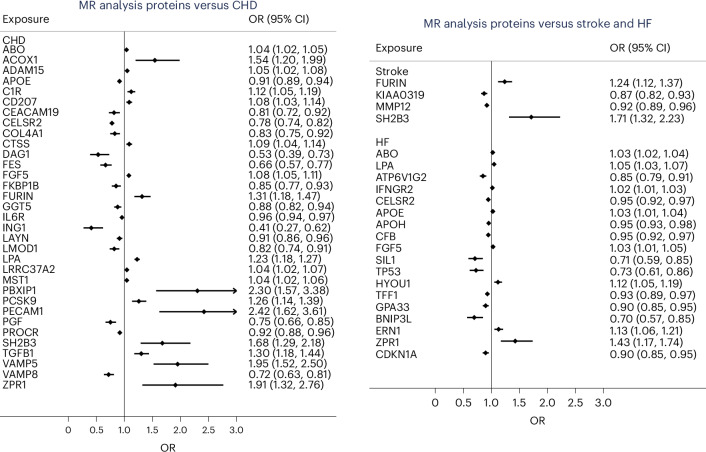
The associations of genetically predicted levels of proteins with the three CVDs. The MR Wald ratio method was used. The results are sorted by the outcome, and only associations with FDR <0.05 (two sided) are shown. The MR estimates are presented corresponding to the change of logarithm of the odds of CVD outcomes per one NPX unit. The diamonds and error bars represent the beta coefficients and 95% CIs, respectively. OR, odds ratio.

As demonstrated in Figs. 1b and 2 and Supplementary Tables 9–11, genetically predicted levels of 33 proteins were related to coronary heart disease (CHD) (at an FDR <0.05) using MR analysis. The top five most strongly associated proteins were LPA, Cadherin EGF LAG seven-pass G-type receptor 2 (CELSR2), APOE, FES and VAMP5. Four proteins were related to IS (MMP12, KIAA0319, FURIN and SH2B3), while 18 were related to HF (the top five being ABO, LPA, ATP6V1G2, IFNGR2 and CELSR2). CELSR2, ABO, LPA, APOE, FGF5 and ZPR1 were related to both CHD and HF, while SH2B3 and FURIN were shared between CHD and stroke on MR analysis. However, not all of these 47 proteins have strong expressions in human heart or artery tissues (Fig. 1c).

Sensitivity analyses using multiple *cis*-single nucelotide polymorphisms (SNPs) on MR analyses showed similar estimates as the Wald ratio estimates of the sentinel *cis*-quantitative trait loci (QTL) SNP for all 47 proteins except for GGT5, ERN1 and CFB (Supplementary Table 12). However, no significant (*P* < 0.05) results were found by any sensitivity analysis for these three proteins. The MR–Steiger test inferred that all of the causal associations were oriented from proteins to the CVD outcomes (Supplementary Table 13).

**Fig. 3:**
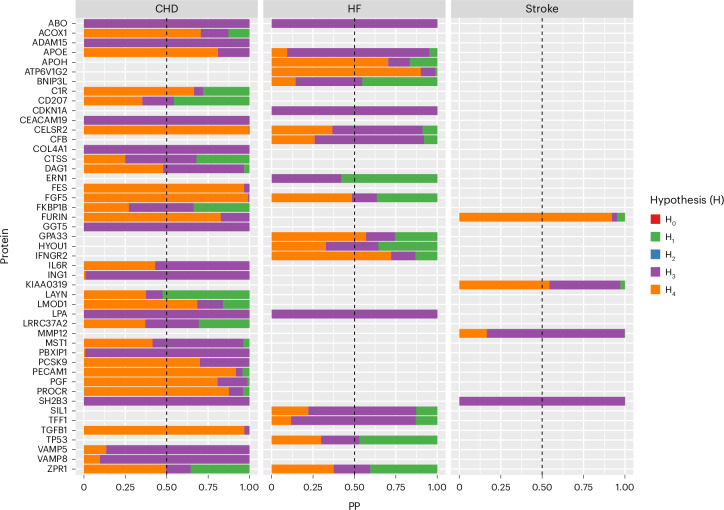
Colocalization analysis of the association of proteins of interest with CVD, including CHD, HF and stroke. H_0_, no traits have a genetic association; H_1_, only protein has a genetic association; H_2_, only CVD has a genetic association; H_3_, protein and CVD both have an association but with different causal variants; and H_4_, protein and CVD are associated with the same causal variant. This Bayesian-based method resulted in five PP to assess the support of each of the five hypotheses. Strong support of colocalization was defined if the PP.H_4_ > 0.8, whereas medium support of colocalization was defined as 0.5 < PP.H_4_ ≤ 0.8. Source data

Using colocalization analysis, 10 of the 47 proteins found in the MR analysis to be linked to a CVD showed a high (>80%) posterior probability (PP) for hypothesis H4, indicating strong evidence to support shared causal variants between proteins and CVD outcomes (CELSR2–CHD and CELSR2–HF; FGF5–CHD; TGFB1–CHD; FES–CHD; FURIN–stroke and FURIN–CHD; PECAM1–CHD; PROCR–CHD; APOE-CHD; PGF–CHD; ATP6V1G2–HF), while eight showed a moderate PP (50–80%) suggesting medium evidence to support colocalization (Fig. 3).

Only 4 of the 47 proteins were significantly associated with CVD on the observational and MR analyses for MI (PGF, LAYN, FURIN and PCSK9). Of those, only FURIN and PCSK9 showed directionally consistent associations on the observational and MR analyses. In total, 6 out of 18 proteins were related to HF on the MR analyses and observational analyses, and only FGF5 and HYOU1 were directionally concordant between both analyses. Of the four proteins that were related to stroke on the MR analysis, only FURIN and MMP12 were also significantly associated with stroke on the observational analyses.

Overall, 30 out of 47 proteins of interest on the MR analyses were identified as potential drug targets, and 10 of those had tier 1 priority, including CTSS, FKBP1B, IL6R, PCSK9, PGF, TGFB1, ERN1, GPA33, IFNGR2 and TP53 (Supplementary Table 14). FGF5, PROCR and FURIN were ranked as tier 3 druggable proteins.

Of the 47 proteins previously linked to at least one of the CVDs on the primary MR analysis, only five were also related to intima-media thickness (IMT) (CEACAM19, PCSK9, APOE, FGF5 and LAYN) (Supplementary Table 15). The corresponding number of proteins was eight for carotid plaque (CELSR2, KIAA0319, DAG1, PGF, FES, TP53, LPA and APOE).

**Fig. 4:**
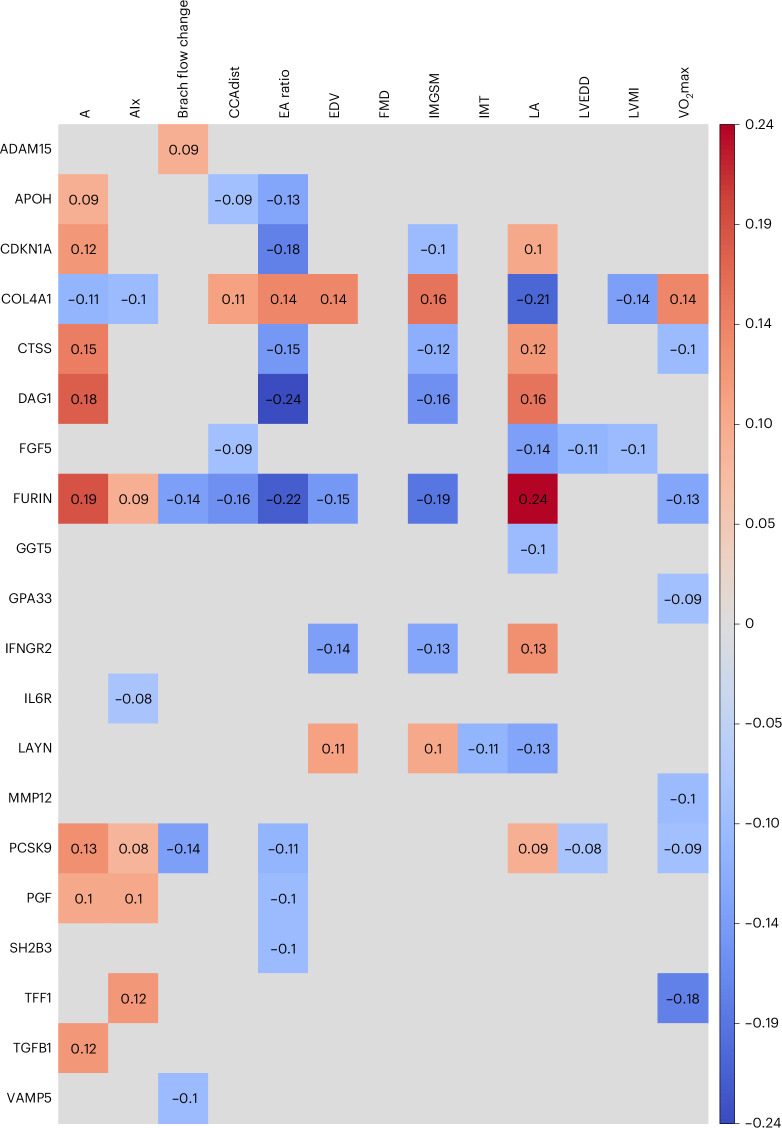
Heat map showing cross-sectional relationships between plasma levels of 20 proteins of interest and subclinical markers of CVDs. Multivariable linear regression was used and the regression coefficients are shown for relationships with *P* < 0.05 (two sided). A gray square indicates *P* ≥ 0.05. A, transmitral A-wave at echocardiography; AIx, augmentation index at radial artery pulse wave analysis; Brach flow change, the increase in brachial artery blood flow following 5 min of blood flow arrest; CCAdist, carotid artery distensibility by ultrasound; EA ratio, left ventricular diastolic function index; EDV, acetylcholine-mediated increase in forearm blood flow; FMD, brachial artery FMD; IMGSM, echolucency of the carotid artery intima–media complex; VO_2_max, maximal oxygen consumption at an exercise test. For complete protein names, see Supplementary Table 16. Source data

In the prospective investigation of obesity and metabolism (POEM) study, plasma levels of 20 out of the 47 proteins of interest were measured. All of those 20 proteins were related to at least one of the markers of subclinical CVD, using *P* < 0.05 (Fig. 4). Plasma levels of COL4A1, CTSS, DAG1, FGF5, FURIN, LAYN, PCSK9 and PGF were related to several of the subclinical markers in the expected direction.

Previous observational studies using the proximity extension assay technique identified GDF15 and TRAILR2 in one study^8^ and GDF15 and WFDC2 in another study to be associated with all three major CVD outcomes^2^. The present much larger study identified 126 proteins levels linked to all three CVD outcomes independently of major confounders, such as age, sex, body mass index (BMI) and glomerular filtration rate^9^. Almost all of those 126 proteins were related to any CVD in the replication phase in CKB and more than half were related to two or three of the CVD. Less than 6% of the proteins related to any CVD in the observational analyses were significant on the MR analysis, suggesting that most observational associations were not causal. This is consistent with recent findings for CHD in the CKB study^10^.

CELSR2 is involved in contact-mediated cell adhesion. *CELSR2* forms a cluster at the 1p13.3 locus with two other genes (*PSRC1* and *SORT1*). A previous MR study indicated a causal role of this cluster for CHD^11^. According to Genotype-Tissue Expression (GTEx), the *cis*-pQTL is expressed in the coronary arteries and aorta. However, a previous study has demonstrated that it is rather a noncoding SNP that creates a transcription factor binding site altering the expression of the *SORT1* gene in the liver. SORT1 in mouse liver had a profound effect on hepatic very-low-density lipoprotein production and thereby low-density lipoprotein (LDL) cholesterol^12^.

PROCR plays a key role in protein C activation and regulation of blood coagulation^13^. A previous MR study suggested a causal role of PROCR in the development of CHD^14^. Currently, two drugs have been registered for targeting PROCR in clinical trials for non-CVD outcomes.

On both observational and MR analyses, FURIN was associated with both MI and IS, which was further supported by colocalization analysis. FURIN is a peptidase known to be involved in the cleavage of precursor proteins to active proteins^15^. According to GTEx, the *cis*-pQTL used as instrument for FURIN is expressed in different arteries, including coronary arteries. A *cis*-pQTL SNP has previously been linked to CHD in genome-wide association studies (GWAS)^16^, in addition to blood pressure^17^ and metabolic syndrome^18^. FURIN is upregulated in the immune cells of human atherosclerotic plaques^19^. Plasma levels of FURIN were also related to several important markers of subclinical CVD in the POEM sample. Plasma FURIN levels have previously been associated with blood pressure, lipids, fasting glucose, obesity and prevalent MI^20^, and was strongly associated with ischemic heart disease (IHD) in the CKB study^10^. According to the database DrugBank, FURIN is currently being explored experimentally as a drug target, but no clinical trials were registered (Supplementary Table 14).

FGF5 is involved in cell signaling. FGF5 was causally linked to IMT and plasma levels of FGF5 in the POEM study were related to a poor carotid artery distensibility. The *cis*-pQTL SNP used as genetic instrument for FGF5 has been linked to CHD^16^ and to blood pressure in previous studies^21,22^.Thus, FGF5 is probably a protein of interest for several CVDs, since hypertension is causally related to all major CVDs^23^.

Only one sentinel *cis*-pQTL of each protein was used to minimize the risk of horizontal pleiotropy^24^, using the approach adopted in UKB^25^. We additionally conducted sensitivity analyses using multiple independent *cis*-pQTLs, but that did only marginally change the MR estimates for most of the proteins.

Only a few proteins identified by the observational analyses showed a significant MR estimate, being directionally consistent with the MR analysis and a high probability of colocalization. The reasons for the discrepant results are probably multifactorial: first, that observational analyses are susceptible to several biases, such as residual confounding and, second, reverse causation. One such example is the N-terminal peptide proBNP and IMT association^6^. Third, the MR estimate is generally interpreted as a life-long effect of the exposure on the outcome^26^, which differs from the limited-time effect estimated by the observational design. Fourth, a complicated phenomenon–developmental compensation could occur if a SNP influences the protein levels during fetal or early postnatal development^27^.

MR can be biased by different, but correlated (in linkage disequilibrium (LD)), causal variants of the exposure and outcome where the latter variant influences the outcome via an alternative pathway^27^. Less than half of the associations found significant by MR showed evidence of colocalization, which is not unusual^28^. For the rest of proteins, the *cis*-pQTLs may be different from the causal variants of CVD or there existed more than one causal variant. The Bayesian colocalization method is also sensitive to the set of SNPs used and the choice of prior probabilities^29^.

The main strength of the present study is the use of the large UKB for both observational and MR analyses. Another strength is the replication in the external CKB. While CKB has protein measurements in a substantial number of incident cases of IHD, it does currently have protein measurements in only a limited number of incident stroke and HF cases. Although the power to reproduce the protein findings in UKB is not optimal, it is reassuring to note that almost all protein findings in UKB were related to at least one of the three CVD outcomes in CKB. Another strength of the present study is that only *cis*-genetic signals for a great number of proteins were used on the MR analyses and colocalization analysis. In addition, we investigated plasma levels for some of the important proteins with several markers of subclinical CVD in POEM to assess the potential mechanisms underlying their associations with CVD outcomes.

**Extended Data Fig. 2:**
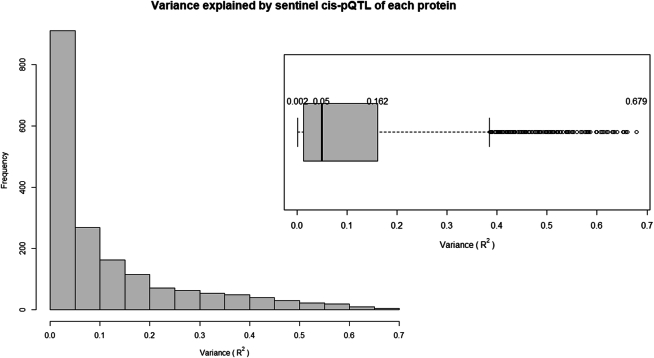
Variance (*R*^2^) of plasma levels of proteins explained by the sentinel cis-pQTL SNPs used as the genetic instrument on the MR analysis. Source data can be found in Supplementary Table 13. The minima is the extreme of the lower whisker (0.002); the extreme of the upper whisker is 0.385; the maxima is rightmost point (0.679); the solid vertical line inside the box represent the median (0.05); the lower and upper hinges of the box represent the first and third quartiles (0.013 and 0.162, respectively).

A limitation is that most of the outcome GWAS studies mainly were conducted in individuals of European descent and therefore the results require validation in other populations. Another limitation is that not all proteins have an identified strong *cis*-SNP. The *cis*-pQTLs used by us explained between 0.2% and 68% of protein-level variance, with a median value of 5% (Extended Data Fig. 2). In a post hoc power calculation (Supplementary Tables 9–11), the power varied greatly for the different proteins and thus false negative findings could occur owing to limited power for some proteins.

Overall, while several hundred proteins were related to CVD, genetically predicted levels of only 47 proteins were linked to CHD, HF or stroke, showing that the vast majority of protein–CVD associations found in observational settings were probably not causal. On the basis of both observational and genetic analyses, several proteins, including FGF5, PROCR and FURIN, are potentially important targets for drug development for CVD prevention.

## Methods

### UKB population sample

The UKB is a large, multicenter, prospective cohort study conducted across the UK. In 2006–2010, over 500,000 individuals aged 40–69 years underwent physical measurements, and blood samples were stored for later analysis of genes and biomarkers. The present study used data from the 52,164 individuals with valid proteomics data. The UKB study was approved by the UK North West Multicentre Research Ethics Committee (application no. 90143) and the Swedish Ethical Review Authority (no. 2023-00148-01), and all participants provided written informed consent.

All participants in UKB had plasma levels of glucose, LDL and high-density lipoprotein (HDL) cholesterol and creatinine measured by a Beckman Coulter AU5800 using standard methods. Blood pressure was measured twice in the sitting position with an automated Omron device. Estimated glomerular filtration rate (eGFR) was calculated by the Chronic Kidney Disease Epidemiology Collaboration (CKD-EPI) formula^30^.

Ethnicity was categorized into four groups: White, Black, Asian and other. The Townsend social deprivation index was used as a marker of socioeconomic status. Smoking status was categorized as never, previous or current cigarette smoking.

All disease outcomes were coded using the 10th edition of the International Classification of Diseases (ICD-10 codes) for first fatal or non-fatal MI (ICD-10 code I21), IS (I63) or HF (I50). These rather narrow definitions of the three CVD outcomes might misclassify some cases as controls, but our previous validation study^31^ indicated that expanding the code range is likely to result in misclassification of a substantial number of cases since additional codes had additional errors. Moreover, minimizing misclassification of cases had a higher priority than minimizing misclassification of controls in this study design.

Plasma levels of 2,923 proteins were measured by the Olink EXPLORE assay (OLINK), which involves a proximity extension assay technique. All assays underwent an extensive quality control evaluation^25^. According to information from the manufacturer, the mean intra-assay and interassay coefficients of variation observed are in the 8–17% range. Assays for four proteins (GLIPR1, NPM1, PCOLCE and CST1) failed quality control in >40,000 individuals, hence the final analyses were limited to 2,919 proteins.

### CKB population sample

CKB is a population-based prospective study of 512,000 Chinese adults aged 30–79 years who were recruited from ten regions (five rural and five urban) in China during 2004–2008. Data were collected on smoking, medical history and education using interview-administered questionnaires. Physical measurements, including BMI and blood pressure, were measured in study clinics. Details of the study design and baseline characteristics have been previously reported^32^. Ethical approval was obtained from the Oxford Tropical Research Ethics Committee, the ethical review committees of the Chinese Center for Disease Control and Prevention, Chinese Academy of Medical Sciences and the institutional review board at Peking University. The Chinese Ministry of Health approved the study at the start in 2004 (including export of data and plasma samples to Oxford), and also approved electronic linkage to health insurance records in 2011. All participants provided written informed consent. Information on incident cases of IHD, IS and HF were obtained from death and disease registers and from the National Health Insurance system for hospital admissions.

Plasma proteins were measured using the Olink EXPLORE panel in stored plasma samples that were collected at baseline in CKB using the same analytical platform as in UKB, with the same number of proteins analyzed.

CKB was used as an external replication dataset for findings in UKB. The sample used in CKB was derived from a nested case–cohort study with 1,937 incident cases of non-fatal or fatal MI or death from IHD (ICD-10 codes: I20, I22–I25) during a 12 year follow-up and a subcohort of 2,001 participants^10^. The mean age was 64 and 51 years and the proportion of women was 38% and 61% in the IHD and subcohort group, respectively. The replication analyses for the present study included 1,937 first incident cases of IHD, 224 cases of IS and 24 cases of HF. For observational analyses of proteins with CVD in CKB, Cox regression models were used to estimate adjusted HRs (and 95% confidence intervals (CIs)) using the Prentice pseudo-partial likelihood for case–cohort designs. All analyses were stratified on the basis of sex and study area, and adjusted sequentially in different models for additional CVD risk factors.

### Two-sample MR

#### Selection of *cis*-pQTLs as genetic instruments

The genetic instruments were obtained from the UKB Pharma Proteomics Project, which is one of the largest genomic studies on protein levels released so far^25^. In this study, pQTLs of 2,923 plasma proteins were discovered in 54,219 UKB participants. We only used the sentinel *cis*-pQTL SNP of each protein (with *P* < 1.7 × 10^−11^) in the combined cohort as genetic instruments if available to limit the chance of horizontal pleiotropy. For proteins with the sentinel *cis*-pQTL being multiallelic variants, we used the meta-analyzed statistics of the discovery (*n* = 34,557) and replication (*n* = 13,358) cohorts if applicable. For proteins with the sentinel *cis*-QTLs being multiallelic in both the combined and the discovery-replication cohorts, we used the SNP within a 1 Mb window of the protein-coding gene that has the lowest *P* value (and required *P* < 5 × 10^−8^ and *F*-statistic > 10) and is biallelic in the combined cohort. A detailed description of the combined and discovery-replication cohorts can be found in ref. ^25^. In total, 1,826 *cis*-pQTL SNPs were retained as instruments (Supplementary Table 16).

#### Data sources for outcomes

We obtained summary-level data for the association of the pQTLs with the three CVD outcomes and carotid artery IMT and carotid plaque from several sources as detailed in Supplementary Table 17.

For CHD, the effect size estimates were obtained from a two-stage meta-analysis study from the CARDIoGRAMplusC4D Consortium, which involved 60,801 CHD cases (mainly MI) and 123,504 controls of European and South and East Asian ancestries^33^. The GWAS summary data of IS was obtained from a multiancestry meta-analysis study of 514,791 individuals, including 60,341 cases and 454,450 controls (MEGASTROKE)^34^. The SNP–HF association estimates were obtained from a multiancestry GWAS meta-analysis including 115,150 cases and 1,550,330 controls^35^.

Summary GWAS results for carotid artery IMT in 71,128 participants and carotid artery plaques in 21,540 out of 48,434 participants examined were obtained from a GWAS of European ancestry populations^36^. Appropriate ethical approvals were obtained from the different samples contributing with data to the different GWAS. The present two-sample MR study was approved by the Swedish Ethical Review Authority.

### Cross-sectional analysis in the POEM study

The POEM study is a population-based cohort of residents of Uppsala City in Sweden, who were aged 50 years (*n* = 502) and included 50% females. All individuals included were of European descent. The data were collected between 2010 and 2016 and further details of data collection and cohort characteristics have been previously reported^37^. The study was approved by the ethics committee of Uppsala University (2009/057), and all participants provided written informed consent.

All participants were investigated after fasting overnight, except the exercise test being performed on another day within a week in the nonfasting state. Blood samples were collected, and plasma samples were stored at −80 °C for later analysis of proteins. A large number of physical tests were performed to obtain information on subclinical markers of CVDs.

#### Bicycle exercise test with gas exchange

Using a bicycle ergonometer, a maximal exercise test with gas exchange measurements was performed (Jaeger Oxygen Pro, Vyaire medical). Blood pressure, heart rate, vital capacity, rate of oxygen consumption (VO_2_) and VCO_2_ were measured at rest, and thereafter the participants were asked to work until exhaustion. The work load was increased by 10 W min^−1^, starting at 30 W for women and 50 W for men. The maximal VO_2_ during the last minute of work was recorded.

#### Blood pressure

Blood pressure was measured manually by a mercury sphygmomanometer in supine position after 30 min of rest. By applying radial pulse wave recordings (see below), central blood pressure was calculated by the software included in the commercial device.

#### The invasive forearm technique

Forearm blood flow was measured by venous occlusion plethysmography (Elektromedicin). After evaluation of resting forearm blood flow, local intra-arterial drug infusions were given in the brachial artery during 5 min for each dose. At the end of each infusion step, forearm blood flow was evaluated. The infused dosages were 25 and 50 µg min^−1^ for acetylcholine (Clin-Alpha) to evaluate endothelium-dependent vasodilation (EDV) in forearm resistance vessels and 5 and 10 µg min^−1^ for SNP (Nitropress, Abbot) to evaluate endothelium-independent vasodilation (EIDV). The two drugs were given in random order between subjects.

EDV in forearm resistance vessels was defined as forearm blood flow during infusion of either 25 or 50 µg min^−1^ of acetylcholine minus resting forearm blood flow divided by resting forearm blood flow (denoted EDV 25 µg and EDV 50 µg). EIDV in forearm resistance vessels was defined as forearm blood flow during infusion of either 5 or 10 µg min^−1^ of SNP minus resting forearm blood flow divided by resting forearm blood flow (denoted EIDV 5 µg or EIDV 10 µg).

#### The brachial artery ultrasound technique

The brachial artery diameter was assessed by external B-mode ultrasound imaging 2–3 cm above the elbow (Acuson XP128 with a 10 MHz linear transducer, Acuson Mountain View). Following measurement of the brachial artery diameter at rest, a blood flow increase was induced by inflation of a pneumatic cuff placed around the forearm to a pressure at least 50 mmHg above systolic blood pressure for 5 min and then a suddenly release of the cuff. Flow-mediated vasodilation (FMD) was defined as the maximal brachial artery diameter recorded between 30 and 90 s following cuff release minus diameter at rest divided by the diameter at rest. Blood flow velocity was recorded by Doppler at rest and in the very early hyperemic period (within 5–10 s). From the Doppler velocity time integral recording, heart rate and the diameter of the vessel, the blood flow was calculated (in millilitres per minute). The blood flow increase during the induced hyperemia was defined as blood flow during hyperemia minus resting blood flow divided by resting blood flow.

#### Pulse wave analysis

A micromanometer tipped probe (Sphygmocor, Pulse Wave Medical Ltd.) was applied to the surface of the skin overlying the radial artery and the peripheral radial pulse wave was continuously recorded. The mean values of around ten pulse waves were used for analyses. On the basis of transfer functions included in the device, aortic systolic and diastolic blood pressures were calculated from the radial recordings. In addition, the augmentation index (AIx), the ratio between the first reflected wave and the systolic peak, was also measured.

#### Carotid artery compliance

The diameter of the common carotid artery of the right side 1–2 cm proximal of the bifurcation was measured by ultrasound M-mode at its maximal diameter in systole and the minimal diameter in diastole (Acuson XP128 with a 10 MHz linear transducer, Mountain View). The distensibility of the carotid artery was calculated as the change in diameter maximum to minimum in relation to the minimal diameter in diastole divided by the central pulse pressure obtained by pulse wave analysis.

#### Carotid artery ultrasound evaluation

The carotid artery was assessed by external B-mode ultrasound imaging (Acuson XP128 with a 10 MHz linear transducer, Mountain View). The IMT was evaluated in the far wall in the common carotid artery 1–2 cm proximal to the bulb.

The images were digitized and imported into the artery measurement software (Artery Measurement Software) automated software for dedicated analysis of IMT and the gray scale median of the intima-media complex. A 10 mm segment with good image quality was chosen for IMT analysis from the carotid artery. The value obtained is the mean of around 100 discrete measurements over the 10 mm segment. The given value for carotid artery IMT is the mean value from both sides.

A region of interest was placed manually around the intima-media segment that was evaluated for IMT, and the program calculates the echogenicity (gray scale) of the intima-media complex from analysis of the individual pixels within the region of interest on a scale from 0 (black) to 256 (white). The blood was used as the reference for black, and the adventitia was the reference for white. The grayscale median value given is the mean value from both sides.

#### Echocardiography and Doppler

A comprehensive two-dimensional and Doppler echocardiography was performed with an Acuson XP124 cardiac ultrasound unit (Acuson) A 2.5 MHz transducer was used for the majority of the examinations.

Left ventricular dimensions were measured with M-mode on-line from the parasternal projections, using a leading-edge to leading-edge convention. Measurements included left atrial diameter (LA), interventricular septal thickness, posterior wall thickness, left ventricular diameter in end diastole (LVEDD) and left ventricular diameter in end systole.

Left ventricular mass (LVM) was determined from the Penn conversion. The LVM was then indexed for height^2.7^ to obtain the left ventricular mass index (LVMI).

The left ventricular diastolic filling pattern of the mitral inflow was obtained from the apical transducer position with the pulsed Doppler sample volume between the tips of the mitral leaflets during diastole. The peak velocity of the early rapid filling wave (E wave) and the peak velocity of atrial filling (A-wave) were recorded, and the E to A ratio (E/A) was calculated.

Means and standard deviations for the markers of subclinical CVD in POEM are given in Supplementary Table 18.

### Statistical analysis

#### Observational analyses

Cox proportional hazards models were used to evaluate whether plasma protein levels were related to incident CVD (combined endpoint of MI, IS or HF) in the primary analysis. Covariates included age, sex, ethnicity, the Townsend deprivation index, smoking (never, previous or current), systolic blood pressure, LDL and HDL cholesterol, diabetes, BMI and eGFR. eGFR was estimated from plasma creatinine using the updated CKD-EPI formula^30^.

One model was run for each protein. In total, 1,832 individuals with prevalent CVD at baseline were excluded from the analyses. The proportional hazard assumption was checked by visual inspection of Kaplan–Meier curves for the proteins of interest. Proteins were analyzed on a *z*-scale, and the HR was expressed as a 1 s.d. change of the normalized protein expression (NPX) unit defined by the manufacturer. These analyses were first performed in a random sample of two-thirds of the complete sample (discovery sample). The proteins showing an FDR Benjamini–Hochberg-adjusted *P* value (shortly as FDR) <0.05 were then evaluated in a similar fashion in the remaining one-third of the complete sample (validation sample). If the associations of these proteins with any one CVD outcome had an FDR <0.05 in the validation sample, such protein–CVD associations were defined as significant. Likewise, we evaluated associations separately with each of the three CVD outcomes (MI, IS and HF). Prevalent cases of the evaluated outcomes were excluded from the respective analyses (1,407 prevalent cases of MI, 196 of IS and 485 of HF). To maximize the power in this part of the study, we used the complete sample, but now required a *P* value <0.000017 (Bonferroni correction) to be statistically significant.

In the replication phase of the 126 proteins related to all three CVD outcomes in UKB, data from CKB were used. Consistent with analyses in UKB, Cox proportional hazards models were used to regress each of those 126 proteins separately with each of the three CVD outcomes. Age, sex, study site, fasting time, education, smoking, physical activity, systolic blood pressure, diabetes, apolipoprotein B/apolipoprotein A ratio, BMI and history of chronic kidney disease were used as covariates. To assess replication, a nominal *P* < 0.05 was regarded as statistically significant.

#### MR analyses

For proteins with *cis*-pQTLs statistics meta-analyzed by those of the discovery and replication cohorts (see ref. ^25^ for details), the MR instrument statistics were generated via a linear fixed-effects model (R package ‘metafor’)^38^ where the weighted estimation with inverse-variance weights was used.

We used the two-sample MR design for the primary analyses (proteins versus the three CVD outcomes). Since only a single *cis*-pQTL SNP was used as the instrument in each MR analysis, the Wald ratio method was applied to obtain the MR estimate using the R package TwoSampleMR^39^. The MR estimates were presented corresponding to the change of logarithm of the odds of binary outcomes or the original unit of continuous outcome (IMT) per one NPX unit, except when stated otherwise. *P* values were adjusted within each outcome using FDR controlling procedure, and <0.05 was deemed statistically significant. The MR analysis of proteins of interest (that is, passing FDR-adjusted *P* < 0.05 threshold in the primary MR analysis) with carotid artery IMT and carotid plaque was conducted in the same manner as the primary analysis. However, this analysis was regarded as exploratory and supportive, and therefore *P* < 0.05 was regarded as statistically significant.

We conducted a post hoc power calculation using the R code provided in the work by Burgess^40^ for the three CVD outcomes. During the calculation, *R*^2^ (variance of exposure explained by the genetic instrument) was estimated using the formula *R*^2^ = beta^2^ × 2 × EAF × (1 − EAF) where beta represents the effect estimate of the SNP–exposure association and EAF is the effect allele frequency^41^.

#### MR sensitivity analyses

We used the MR–Steiger test to examine whether the estimated effect was correctly oriented from proteins to CVD outcomes. For the proteins of interest, we further performed sensitivity analyses including MR inverse-variance weights, MR weighted median, MR-weighted mode and MR Egger. In brief, independent (low LD, defined as *R*^2^ < 0.01, clumping window >10 kb) GWAS-significant (*P* < 5×10^−8^) SNPs at the *cis*-loci (within 1 Mb) of each protein-coding gene with *F*-statistic >10 were selected as instruments. Horizontal pleiotropy (*P* value of MR-Egger intercept) and heterogeneity (Cochrane’s *Q* value) of the instruments were examined by the relevant functions integrated in the R package TwoSampleMR. A total of 13 of 47 proteins of interest had a single independent *cis*-SNP at the defined window, and therefore no sensitivity analyses were conducted for these proteins.

#### Colocalization analysis

To identify whether the MR associations of proteins of interest with CVD outcomes were driven by LD, we further performed colocalization analysis using the R package Coloc^29^. The analysis was based on the enumeration method to gauge the support for five exclusive hypotheses regarding two potentially related traits (T1 and T2) in a predefined genomic region: (1) H_0_: no traits have a genetic association; (2) H_1_: only T1 has a genetic association; (3) H_2_: only T2 has a genetic association; (4) H_3_: T1 and T2 both have an association but with different causal variants; and (5) H_4_: T1 and T2 are associated with the same causal variant. This Bayesian-based method results in five PP to assess the support of each of the five hypotheses. In this analysis, three prior probabilities were set as follows: p1 (a SNP is associated with T1) = 1 × 10^–4^; p2 (a SNP is associated with T2) = 1 × 10^–4^; for p12 (a SNP is associated with both traits), we used a more conservative value, 5 × 10^−6^, as suggested previously for improved robustness^42^. The sensitivity of colocalization inference to variations in p12 was further examined by visualization. Strong support of colocalization was defined if the PP.H_4_ >0.8, whereas medium support of colocalization was defined as 0.5 < PP.H_4_ ≤ 0.8.

#### Pathway enrichment and GTEx analyses

To determine whether CVD-associated proteins were enriched in specific pathways, an overrepresentation analysis was conducted at Reactome^43^ for all proteins associated with the combined CVD endpoint in the observational setting. For the proteins of interest found on MR analysis, the expression level (transcript per million) of their coding genes in human heart and artery tissues was further looked up on the GTEx Portal.

#### Protein druggability

For proteins of interest, we sought evidence for druggability tiers using the approach previously reported by Finan et al.^44^, where tier 1 was defined as proteins targeted by approved drugs and drugs in clinical development, tier 2 as proteins closely related to drug targets or with associated drug-like compounds and tier 3 as extracellular proteins and members of key drug target families. For each protein, detailed targeting drug information was curated from DrugBank, clinicaltrials.gov and ChEMBL databases wherever available.

#### Cross-sectional evaluation in the POEM study

Plasma levels of the proteins found to be of interest in the MR analyses were inverse-normalized rank-transformed and were thereafter associated with each of the subclinical markers of CVD in the POEM sample using linear regression models, including sex as a covariate (age was the same in all subjects). *P* < 0.05 was considered statistically significant in this supportive analysis.

All the observational analyses were conducted in STATA (version 16.1), while the MR and colocalization analyses were conducted in R (version 4.1.0). All statistical tests were two tailed.

### Reporting summary

Further information on research design is available in the Nature Portfolio Reporting Summary linked to this article.

## Supplementary information

Reporting Summary

Supplementary Tables 1–18 Supplementary Tables 1–18.

## Source data

Source Data Fig. 3 Statistical source data.

Source Data Fig. 4 Statistical source data.

## Figures and Tables

**Extended Data Table 1:**
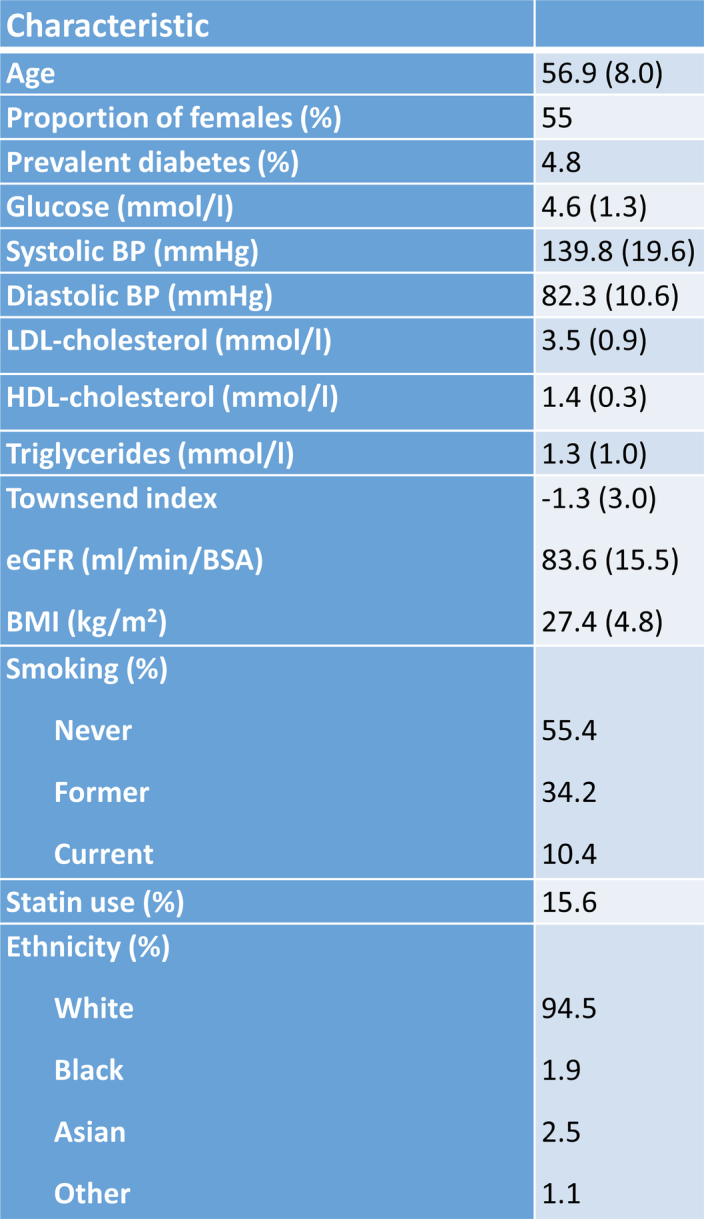
Basic characteristics of the UK Biobank participants with protein measurements

Means and SD or proportions are given (*n* = 52,164).

## Data Availability

The UKB and its data are an open research resource, available following submission of a research plan at https://www.ukbiobank.ac.uk. The CKB observational data that support the findings of this study are available to bona fide researchers on application under the CKB Open Access Data Policy (www.ckbiobank.org). Summary-level GWAS data are available for proteins at https://metabolomips.org/ukbbpgwas/, for CHD at http://www.cardiogramplusc4d.org/, for IS at https://www.megastroke.org/, for HF at https://www.ebi.ac.uk/gwas/studies/GCST90162626 and for ultrasound-measured carotid artery IMT and carotid plaques at https://www.ncbi.nlm.nih.gov/projects/gap/cgi-bin/study.cgi?study_id=phs000930.v6.p1 (accession no. phs000930.v6.p1). Data supporting the findings of the POEM study are provided in the article and related files. Raw data are not publicly available owing to Swedish law, as they contain sensitive personal information, but could be obtained from the POEM study following a request to lars.lind@medsci.uu.se. Other online databases used are Reactome (https://reactome.org/, version 86 on 03/11/2023), GTEx Portal (https://gtexportal.org/home, dbGaP accession no. phs000424.v8.p2), DrugBank (https://go.drugbank.com/), clinical trials (https://clinicaltrials.gov/) and ChEMBL (https://www.ebi.ac.uk/chembl/). Source data are provided with this paper.

## Code availability

The code used for the main analyses in this work is available via Zenodo at 10.5281/zenodo.13347408 (ref. ^45^).
