# Supplementary material for: Measured and genetically predicted protein levels and cardiovascular diseases in UK Biobank and China Kadoorie Biobank
Source: Nat Cardiovasc Res. 2024 Sep 25;3(10):1189–98. doi: 10.1038/s44161-024-00545-6 (PMC11473359; doi:10.1038/s44161-024-00545-6)
Supplement: Supplementary file 1 — Reporting Summary [file 44161_2024_545_MOESM1_ESM.pdf]

nature portfolio

Corresponding author(s): Rui Zheng

Last updated by author(s): Aug 19, 2024

Reporting Summary

Nature Portfolio wishes to improve the reproducibility of the work that we publish. This form provides structure for consistency and transparency in reporting. For further information on Nature Portfolio policies, see our Editorial Policies and the Editorial Policy Checklist.

Statistics

For all statistical analyses, confirm that the following items are present in the figure legend, table legend, main text, or Methods section.

n/a	Confirmed	
☐	☒	The exact sample size (*n*) for each experimental group/condition, given as a discrete number and unit of measurement
☒	☐	A statement on whether measurements were taken from distinct samples or whether the same sample was measured repeatedly
☐	☒	The statistical test(s) used AND whether they are one- or two-sided<br>*Only common tests should be described solely by name; describe more complex techniques in the Methods section.*
☐	☒	A description of all covariates tested
☐	☒	A description of any assumptions or corrections, such as tests of normality and adjustment for multiple comparisons
☐	☒	A full description of the statistical parameters including central tendency (e.g. means) or other basic estimates (e.g. regression coefficient) AND variation (e.g. standard deviation) or associated estimates of uncertainty (e.g. confidence intervals)
☐	☒	For null hypothesis testing, the test statistic (e.g. *F*, *t*, *r*) with confidence intervals, effect sizes, degrees of freedom and *P* value noted<br>*Give P values as exact values whenever suitable.*
☐	☒	For Bayesian analysis, information on the choice of priors and Markov chain Monte Carlo settings
☒	☐	For hierarchical and complex designs, identification of the appropriate level for tests and full reporting of outcomes
☐	☒	Estimates of effect sizes (e.g. Cohen's *d*, Pearson's *r*), indicating how they were calculated

Our web collection on statistics for biologists contains articles on many of the points above.

Software and code

Policy information about availability of computer code

Data collection	No specific softwares were used.
Data analysis	All the analyses for the observational part were conducted in STATA (version 16.1); the MR and colocalization were conducted in R (version 4.1.0). All core functions used for data analysis are integrated in the respective packages.<br>*R packages:<br>Metafor, version 3.4.0;<br>TwoSampleMR, version 0.5.6;<br>Coloc, version 5.1.0.1.

For manuscripts utilizing custom algorithms or software that are central to the research but not yet described in published literature, software must be made available to editors and reviewers. We strongly encourage code deposition in a community repository (e.g. GitHub). See the Nature Portfolio guidelines for submitting code & software for further information.

Data

Policy information about availability of data

All manuscripts must include a data availability statement. This statement should provide the following information, where applicable:

- Accession codes, unique identifiers, or web links for publicly available datasets
- A description of any restrictions on data availability
- For clinical datasets or third party data, please ensure that the statement adheres to our policy

I. The UK Biobank and its data is an open research resource available following submission of a research plan at https://www.ukbiobank.ac.uk.
II. The CKB observational data that support the findings of this study are available to bona fide researchers on application under the China Kadoorie Biobank Open Access Data Policy (www.ckbiobank.org).
III. Summary-level GWAS data of
• proteins available at https://metabolomips.org/ukbbpgwas/.
• coronary heart disease (CHD) available at http://www.cardiogramplusc4d.org/.
• ischemic stroke available at https://www.megastroke.org/.
• heart failure (HF) available at https://www.ebi.ac.uk/gwas/studies/GCST90162626.
• ultrasound-measured carotid artery intima-media thickness (IMT) and carotid plaques available at https://www.ncbi.nlm.nih.gov/projects/gap/cgi-bin/study.cgi?study_id=phs000930.v6.p1; accession phs000930.v6.p1.
IV. Data supporting the findings of the POEM study are provided in the article and related files. Raw data are not publicly available due to Swedish law, as they contain sensitive personal information, but could be obtained from the POEM study following a request to: lars.lind@medsci.uu.se).
V. Other online datasets
*Reactome (https://reactome.org/), version 86 on 03/11/2023 for pathway enrichment analysis.
*GTEx Portal (https://gtexportal.org/home, dbGaP accession number phs000424.v8.p2) for the look-up of expression levels of protein-coding genes in several human tissues.
*DrugBank (https://go.drugbank.com/), https://clinicaltrials.gov/ and ChEMBL (https://www.ebi.ac.uk/chembl/) databases for druggability look-up.

Research involving human participants, their data, or biological material

Policy information about studies with human participants or human data. See also policy information about sex, gender (identity/presentation), and sexual orientation and race, ethnicity and racism.

Reporting on sex and gender	Both sexes were included in the cohort populations to represent the general population. Sex information was collected in each cohort. Data have been collected stratified on sex to maximize statistical efficiency. No information of gender was collected.
Reporting on race, ethnicity, or other socially relevant groupings	This study used data from multiple cohorts with multiple ancestries. Detailed information can be found in the cited references.
Population characteristics	The UKB study: UKB is a large, multi-center, prospective cohort study conducted across the UK. Over 500,000 individuals aged 40–69years were included.<br><br>The CKB study: CKB is a population-based prospective study of 512,000 Chinese adults aged 30-79 years.<br><br>The POEM study: inhabitants of the city of Uppsala, Sweden, all aged 50 years (n=502).
Recruitment	The UKB study: In 2006–10, over 500,000 individuals aged 40–69years underwent physical measurements, and blood samples were stored for later analysis of genes and biomarkers. The present study used data from the 52,164 individuals with valid proteomics data. Detailed information can be found at https://www.ukbiobank.ac.uk.<br><br>The CKB study: Participants were recruited from 10 regions (5 rural and 5 urban) in China during 2004-2008. Detailed description can be found from a previous work: Chen Z et al. China Kadoorie Biobank of 0.5 million people: survey methods, baseline characteristics and long-term follow-up. Int J Epidemiol. 2011;40(6):1652-66.<br><br>The POEM study: Information from our previous publication "Relationships between three different tests to evaluate endothelium-dependent vasodilation and cardiovascular risk in a middle-aged sample" by Lars Lind (DOI: 10.1097/HJH.0b013e3283619d50):<br>The individuals were invited in a random order from the register of inhabitants in the city 1 month following their 50th birthday. No exclusion criteria were applied except that the individuals needed to have a Swedish identification number.
Ethics oversight	The UK Biobank study was approved by the UK North West Multi-Centre Research Ethics Committee (Application Nr. 90143) and the Swedish Ethical Review Authority (Nr. 2023-00148-01).<br><br>Ethical approval for CKB was obtained from the Oxford Tropical Research Ethics Committee, the Ethical Review Committees of the Chinese Center for Disease Control and Prevention, Chinese Academy of Medical Sciences, and the Institutional Review Board (IRB) at Peking University.

The POEM study was approved by the Ethics committee of Uppsala University (2009/057) .

Note that full information on the approval of the study protocol must also be provided in the manuscript.

Field-specific reporting

Please select the one below that is the best fit for your research. If you are not sure, read the appropriate sections before making your selection.

☒ Life sciences ☐ Behavioural & social sciences ☐ Ecological, evolutionary & environmental sciences

For a reference copy of the document with all sections, see nature.com/documents/nr-reporting-summary-flat.pdf

Life sciences study design

All studies must disclose on these points even when the disclosure is negative.

Sample size	For the observational analysis, 52,164 individuals from the UK Biobank and 502 individuals from POEM study, and 3938 participants from CKB study. Post-hoc power calculation was conducted for the MR analysis.
Data exclusions	Protein data failed quality control were excluded and data of individuals with prevalent CVDs were excluded in the observational analysis. Proteins with no cis-pQTLs or no rsIDs and pQTLs being multiallelic genetic variants were excluded in the MR and colocalization analyses.
Replication	The CKB study was used as an independent external replication of the observed protein-CVD associations. Only one replication was conducted. A total of 126 of the proteins were related to all three CVD outcomes in UK Biobank and of those, 118 were related to any of the CVD traits in the replication phase in CKB.
Randomization	Only observational design was used for our study and thus randomization was not required.
Blinding	Irrelevant. All information was collected before proteins were analyzed. Protein instrumental analysis was automated.

Reporting for specific materials, systems and methods

We require information from authors about some types of materials, experimental systems and methods used in many studies. Here, indicate whether each material, system or method listed is relevant to your study. If you are not sure if a list item applies to your research, read the appropriate section before selecting a response.

Materials & experimental systems

n/a	Involved in the study	
☒	☐	Antibodies
☒	☐	Eukaryotic cell lines
☒	☐	Palaeontology and archaeology
☒	☐	Animals and other organisms
☒	☐	Clinical data
☒	☐	Dual use research of concern
☒	☐	Plants

Methods

n/a	Involved in the study	
☒	☐	ChIP-seq
☒	☐	Flow cytometry
☒	☐	MRI-based neuroimaging

Plants

Seed stocks	*Report on the source of all seed stocks or other plant material used. If applicable, state the seed stock centre and catalogue number. If plant specimens were collected from the field, describe the collection location, date and sampling procedures.*
Novel plant genotypes	*Describe the methods by which all novel plant genotypes were produced. This includes those generated by transgenic approaches, gene editing, chemical/radiation-based mutagenesis and hybridization. For transgenic lines, describe the transformation method, the number of independent lines analyzed and the generation upon which experiments were performed. For gene-edited lines, describe the editor used, the endogenous sequence targeted for editing, the targeting guide RNA sequence (if applicable) and how the editor was applied.*
Authentication	*Describe any authentication procedures for each seed stock used or novel genotype generated. Describe any experiments used to assess the effect of a mutation and, where applicable, how potential secondary effects (e.g. second site T-DNA insertions, mosiacism, off-target gene editing) were examined.*
